# JMV5656, A Novel Derivative of TLQP-21, Triggers the Activation of a Calcium-Dependent Potassium Outward Current in Microglial Cells

**DOI:** 10.3389/fncel.2017.00041

**Published:** 2017-02-23

**Authors:** Ilaria Rivolta, Anna Binda, Laura Molteni, Laura Rizzi, Elena Bresciani, Roberta Possenti, Jean-Alain Fehrentz, Pascal Verdié, Jean Martinez, Robert J. Omeljaniuk, Vittorio Locatelli, Antonio Torsello

**Affiliations:** ^1^Department of Medicine and Surgery, University of Milano-BicoccaMonza, Italy; ^2^Department of Medicine of Systems, University of Rome “Tor Vergata”Rome, Italy; ^3^CNRS, Institut des Biomolécules Max Mousseron UMR5247, École Nationale Supérieure de Chimie de Montpellier – University of MontpellierMontpellier, France; ^4^Department of Biology, Lakehead University, Thunder BayON, Canada

**Keywords:** TLQP-21, microglia, patch clamp, Ca^2+^-activated K^+^ channels, K^+^ current

## Abstract

TLQP-21 (TLQPPASSRRRHFHHALPPAR) is a multifunctional peptide that is involved in the control of physiological functions, including feeding, reproduction, stress responsiveness, and general homeostasis. Despite the huge interest in TLQP-21 biological activity, very little is known about its intracellular mechanisms of action. In microglial cells, TLQP-21 stimulates increases of intracellular Ca^2+^ that may activate functions, including proliferation, migration, phagocytosis and production of inflammatory molecules. Our aim was to investigate whether JMV5656 (RRRHFHHALPPAR), a novel short analogue of TLQP-21, stimulates intracellular Ca^2+^ in the N9 microglia cells, and whether this Ca^2+^ elevation is coupled with the activation Ca^2+^-sensitive K^+^ channels. TLQP-21 and JMV5656 induced a sharp, dose-dependent increment in intracellular calcium. In 77% of cells, JMV5656 also caused an increase in the total outward currents, which was blunted by TEA (tetraethyl ammonium chloride), a non-selective blocker of voltage-dependent and Ca^2+^-activated potassium (K^+^) channels. Moreover, the effects of ion channel blockers charybdotoxin and iberiotoxin, suggested that multiple calcium-activated K^+^ channel types drove the outward current stimulated by JMV5656. Additionally, inhibition of JMV5656-stimulated outward currents by NS6180 (4-[[3-(trifluoromethyl)phenyl]methyl]-2H-1,4 benzothiazin-3(4H)-one) and TRAM-34 (triarylmethane-34), indicated that K_Ca_3.1 channels are involved in this JMV5656 mechanisms of action. In summary, we demonstrate that, in N9 microglia cells, the interaction of JMV5656 with the TLQP-21 receptors induced an increase in intracellular Ca^2+^, and, following extracellular Ca^2+^ entry, the opening of K_Ca_3.1 channels.

## Introduction

Microglial cells play a pivotal role in the first line of host defense in the central nervous system (Plata-Salaman, 1991). Activated microglia can secrete pro-inflammatory and pro-nociceptive mediators including, but not limited to, tumor necrosis factor (TNF), interleukins 1β, 6, 10, and 18 (IL-1β, IL-6, IL-10, and IL-18), and brain-derived growth factor (BDNF), all of which may be involved in the pathogenesis of chronic and neuropathic pain (Opree and Kress, 2000; Coull et al., 2005; Berta et al., 2014). Neuropathic pain, developing as the result of central or peripheral nervous system damages, is an important clinical problem. Comparison of gene expression profiles in primary sensory neurons following various insults has shown that VGF (non-acronymic) expression was always up-regulated (Maratou et al., 2009). VGF mRNA is expressed throughout the central and the peripheral nervous system. In the rat, the highest levels of VGF mRNA have been measured in the arcuate nucleus of the hypothalamus and in the granular layer of the cerebellum (Snyder and Salton, 1998). VGF is a member of the extended granin family (Bartolomucci et al., 2011), comprising acidic ubiquitous proteins present in secretory cells of the nervous, endocrine and immune systems. It is a propeptide of 617 amino acids in length in mouse and rat, 615 amino acids in human with a highly conserved sequence presenting only variation of 1–2 amino acids in different species. It is processed by PC1/3 and PC2 prohormone convertases to yield several bioactive peptides (Levi et al., 2004). VGF and peptides derived from its processing have been found in dense core vesicles and are released from neuronal and neuroendocrine cells via the regulated secretory pathway (Possenti et al., 1999). TLQP-21 (VGF^556-576^) is one of most studied among VGF-derived neuropeptides, it is a multifunctional protein which modulates lipolysis, energy balance, gonadotropin, and insulin release, as well as gastroenteric functions and inflammatory pain (Bartolomucci et al., 2006; Severini et al., 2009; Pinilla et al., 2011; Possenti et al., 2012). As far as concerned its action on microglial cells, it has been reported that the activity of C3AR1, a receptor of TLQP-21 that is expressed in microglia, has been implicated in a spectrum of immunomodulatory processes; moreover, TLQP-21 seems to induce p38 MAP kinase phosphorylation and this activation is related to the release of prostaglandins from microglia. Finally, TLQP-21 may lead to production and secretion of some cytokines through gC1qR (Chen et al., 2013; Fairbanks et al., 2014). Although these biological activities have stimulated enormous investigational interest, very little is known about the mechanism of TLQP-21 action at the cellular level. TLQP-21 displays saturable binding to adipocyte membranes and atomic force microscopy demonstrated the expression of a single class of binding sites by CHO cells (Possenti et al., 2012; Cassina et al., 2013). It has been reported that, TLQP-21 induces an increase in intracellular calcium [Ca^2+^]_i_ levels in ovary, microglial and pituitary cells (Cassina et al., 2013; Chen et al., 2013; Petrocchi Passeri et al., 2013), probably by mobilizing thapsigargin-sensitive stores. In microglial cells, the resident macrophages of the brain, the organization of intracellular Ca^2+^ signals results from tightly coordinated fluxes of Ca^2+^ through intracellular and plasmalemmal membranes (Verkhratsky and Parpura, 2014). This implies a very precise coupling of the mechanisms regulating intracellular calcium homeostasis with external stimuli involving the consequent opening of Ca^2+^-activated potassium (K^+^) channels. These potassium channels play a central role in several microglial functions, including activation, respiratory burst, proliferation, migration, phagocytosis, and production of inflammatory molecules (Ohana et al., 2009; D’Alessandro et al., 2013). Calcium activated potassium channels are a heterogeneous family and are subgrouped on the basis of conductance as large- (BK or K_Ca_1.1), small- (SK or K_Ca_2.3) and intermediate- (IK or K_Ca_3.1) conductance K_Ca_ channels. In principle, they provide a polarizing and potassium extrusion-influence which is integral to regulation of intracellular calcium homeostasis. It has been reported that microglial cells express all the three families of these calcium dependent channels (Kaushal et al., 2007; Schlichter et al., 2010). Interestingly, the elevation in intracellular Ca^2+^ levels is not sufficient to activate K_Ca_3.1 and K_Ca_2.3 in microglia MLS-9 cells, whereas riluzole, a neuroprotective drug, can activate both channels without inducing significant Ca^2+^ elevations (Ferreira and Schlichter, 2013).

The purpose of this study was to investigate whether JMV5656 (TLQP-21^9-21^), a novel short analog of TLQP-21, can stimulate an increase in intracellular Ca^2+^ in the murine microglial cell line N9, and whether this Ca^2+^ elevation is coupled with the activation of Ca^2+^-dependent K^+^ channels.

## Materials and Methods

### Chemicals

TLQP-21 (TLQPPASSRRRHFHHALPPAR) and JMV5656 (RRRHFHHALPPAR) were synthesized by conventional solid phase peptide synthesis and then purified on a C18 reversed phase column. Each peptide was purified to a purity of at least 95% by high-performance liquid chromatography (chromatograms are shown in **Supplementary Figure S1**). Unless specified, all other reagents were from Sigma–Aldrich (St Louis, MO, USA).

### Cell Cultures

The murine microglial N9 cells (Corradin et al., 1993) were grown in Iscove’s Modified Dulbecco’s Medium (IMDM; Sigma) supplemented with 5% heat-inactivated fetal bovine serum (FBS), 2 mM L-glutamine, 100 IU/ml penicillin, 100 μg/ml streptomycin (Euroclone, Pero, Italy) and cultured in a controlled environment (at 37°C in humidified incubator with 5% CO_2_). Stock cells were passaged 2–3 times/week with 1:10 split ratio and used within eight passages.

### Intracellular Ca^2+^ (Mobilization Assay)

N9 cells were plated at 20,000 cells/well into black walled, clear bottom 96-well plate (Greiner Bio One, Kremsmünster, Austria) and cultured two days up to 90-100% of confluence. Prior to assay, cells were incubated in darkness with 100 μl of HBSS solution containing 20 mM HEPES, 2.5 mM probenecid and 4.5 μM FLUO-4 NW (Molecular Probes, Eugene, OR, USA) at 37°C and 5% CO_2_ for 40 min. Probenecid is commonly used to inhibit organic-anion transporters located in the cell membrane to minimize the leakage of the intracellular dye back to the extracellular environment. Fluorescence emissions were measured with the multi-label spectrophotometer VICTOR3 (Perkin Elmer, MA, USA) at 485/535 nm (excitation/emission filters) every 0.5 s for the 20 s preceding and for the 60 s following peptide exposure. TLQP-21 and JMV5656 (1 nM–10 μM) were dissolved in HBSS and injected into the wells by an automated injector system. Fluorescence data have been calculated as (maximum fluorescence–basal fluorescence)/basal fluorescence (ΔF/F), or the percent increase from the last value before stimulation ((Fs/F_0_)^∗^100) where F_0_ is the last basal value before stimulation and Fs is the stimulated value. To assure reproducibility, each experiment has been repeated three times in different days. To control for cell viability and compare the magnitude of the stimulation achieved by TLQP-21 and JMV5656, cells were also stimulated with 10 μM ATP dissolved in HBSS.

### Electrophysiological Recordings

For electrophysiological recordings, N9 cells were plated in p35 dishes (BD Falcon, Sacco, Milano, Italy) 24 h before patch-clamp experiments and cultured in a controlled environment. Just before the current measurements, culture medium was replaced with an extracellular solution previously reported containing 135 mM NaCl, 5.4 mM KCl, 1.8 mM CaCl_2_, 1 mM MgCl_2_, 0.4 mM NaH_2_PO_4_, 10 mM HEPES, 10 mM glucose (Vecchietti et al., 2006). Whole-cell patch-clamp recordings were performed at room temperature (RT) using pipette pulled to a resistance of 2–5 MΩ (Model P-97 Sutter Instruments, Novato, CA, USA). If not otherwise stated, the pipette intracellular solution contained 122 mM KAsp, 20 mM KCl, 1 mM MgCl_2_, 1.6 mM CaCl_2_, 10 mM HEPES, 5 mM EGTA. In this condition the free intracellular Ca^2+^ concentration was 103,2 nM^1^. In experiments that required intracellular free calcium concentration of 3 μM, EGTA was lowered to 1.8 and CaCl_2_ increased to 1.7 mM. In some experiments, EGTA was replaced with 5 mM BAPTA as alternative calcium chelator. In each of these circumstances the osmolarity was adjusted accordingly. Recordings were made with a Multiclamp 700B amplifier, and data were digitized with a Digidata 1440A and pClamp 10.3 software (all from Axon Instruments, Molecular Devices, Sunnyvale, CA, USA). Results were analyzed with Clampfit 10.3 software.

Before studying the effects of JMV5656 on ionic currents in N9 cells, we tested whether the shear stress caused by the superfusion itself could evoke an electrical activity of stretch-activated channels. When stimulated with a step protocol of 400 ms duration, ranging from –120 to +60 mV every 5 s for up to 5 min, from a holding potential of –80 mV, N9 cells did not exhibit differences in the total transmembrane currents upon the superfusion with the extracellular solution (*n* = 8; data not shown). These experiments confirmed that no stretch-activated channels were activated under these conditions. Therefore, superfused N9 cells could be considered as a suitable model to study the electrophysiological effects of the peptide.

JMV5656 and channel blockers tetraethyl ammonium chloride (TEA), 4-aminopyridine (4-AP), apamin, charybdotoxin (CTX), iberiotoxin (IbTX), triarylmethane-34 (TRAM-34), NS6180 were added to the bath solution. Extracellular solution without calcium contained 135 mM NaCl, 5.4 mM KCl, 1.8 mM EGTA, 1 mM MgCl_2_, 0.4 mM NaH_2_PO_4_, 10 mM HEPES, 10 mM glucose.

Every condition was tested as follow: the extracellular solution containing any given channel blocker was superfused for a minimum of 15 s. After the membrane currents had reached a new equilibrium due to the effect of the perfusion (defined as basal current), we applied the extracellular solution containing the selected channel blocker and 10 μM JMV5656.

### Silencing of K_Ca_3.1 with siRNA

For mRNA silencing we used specific K_Ca_3.1 siRNA duplex (sense: 5′-CGGAGAAACACGUGCACAAdTdT-3′; antisense: 5′-UUGUGCACGUGUUUCUCCGdTdT-3′) (Eurofins Genomics; Vimodrone, Italy). To control for transfection non-specific effects the negative control group was transfected with C3AR1 siRNA (sense: 5′-GUGUACCAGUAUUUGUAUAdTdT-3′; antisense: 5′-UAUACAAAUACUGGUACACdTdT-3′) (Eurofins Genomics). Transfection was performed in a 24-well plate (Euroclone) using DharmaFECT 1 Transfection Reagent (Thermo Scientific, Lafayette, CO, USA) according to the manufacturer’s protocol. Subsequent experiments were performed 24 h after transfection.

### PCR

Total RNA was extracted from N9 cells using EuroGOLD Trifast reagent (Euroclone). For each sample 160 ng of total RNA were transcribed to cDNA using M-MLV Reverse Transcriptase (Invitrogen, Waltham, MA, USA). cDNA was amplified by PCR using GoTaq^®^ G2 DNA Polymerase (Promega, Madison, WI, USA) and the following primers (Sigma): mouse K_Ca_3.1 forward: 5′-CTGAGAGGCAGGCTGTCAATG-3′; mouse K_Ca_3.1 reverse: 5′-ACGTGTTTCTCCGCCTTGTT-3′; GAPDH forward: 5′-GCCATCAACGACCCCTTCATTG-3′; GAPDH reverse: 5′-TCTGTCATGAGGTTGGCTTTCAG-3′.

### Statistical Analysis

Values are expressed as mean ± SE. The statistical significance of differences between groups was evaluated with two-tailed Student’s *t*-test or, when appropriate, by one-way analysis of variance (ANOVA) followed by or Kruskal-Wallis test. A *p*-value of less than 0.05 was considered statistically significant and indicated with ^∗^ in the figures.

## Results

### JMV5656 Stimulates Intracellular Calcium Mobilization in N9 Cells

**FIGURE 1:**
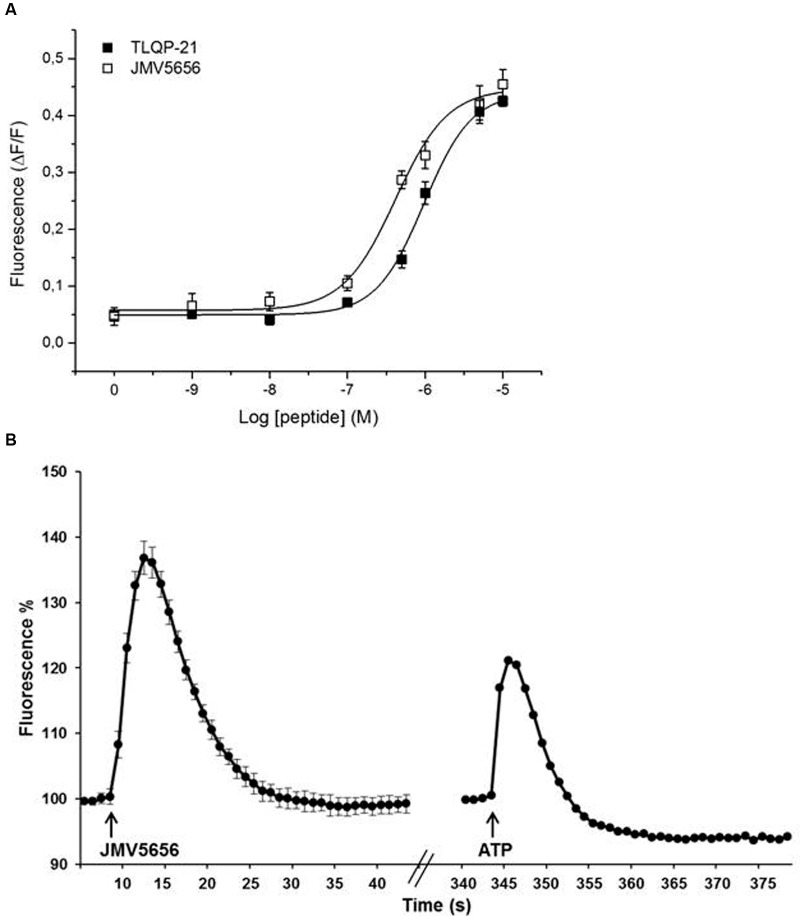
**Effect of TLQP-21 and JMV5656 on calcium mobilization in N9 cells.** **(A)** Dose-response study of intracellular calcium stimulation by TLQP-21 and JMV5656. Intracellular calcium concentrations were measured in N9 cells using the fluorophore Fluo-4 NW as indicated in the Section “Materials and Methods”. Data represent the peak values of intracellular calcium levels achieved for each concentration of the stimuli, and were calculated as ΔF/F, meaning (maximum fluorescence–basal fluorescence)/basal fluorescence. The 0 concentration is the ΔF/F measured in cells stimulated with the vehicle only; the small increase in fluorescence is likely induced by the injection procedure itself. Each point is the mean ± SE of 18 measurements obtained in three independent experiments. **(B)** A representative time-course of intracellular calcium stimulation by JMV5656. Data were calculated as Fluorescence %, meaning (fluorescence after stimulation/last basal fluorescence before stimulation)^∗^100. The effects of 10 μM ATP is shown for comparison. Arrows indicate injection of the stimuli. Fluorescence emissions were measured at 485/535 nm (excitation/emission filters) every 0.5 s for the 20 s preceding and for the 60 s following peptide as indicated in the Section “Materials and Methods”.

We have performed dose-response studies (1 nM–10 μM) to test the activity of TLQP-21 and JMV5656 on N9 cells. Both peptides induced a dose-dependent acute increase in intracellular calcium (**Figure 1A**). The concentrations in the 0.1–10 μM range evoked a significant increase in intracellular calcium levels in N9 cells, reaching a plateau at the higher doses. Interestingly, JMV5656 was slightly more potent in inducing a calcium response than TLQP-21 (EC_50_ TLQP-21: 0.96 μM vs. EC_50_ JMV5656: 0.45 μM). The kinetic of calcium increase after stimulation showed that the peak levels of intracellular calcium increase were reached in 3–4 s, and basal levels restored within 20 s from stimulation (**Figure 1B** and **Supplementary Figure S2**). Interestingly, 10 μM JMV5656 stimulated intracellular calcium rise with a kinetic comparable to that of ATP 10 μM, but JMV5656 appeared more effective (**Figure 1B**): about 37% increase in presence of JMV5656 and 20% in presence of ATP. Since it has been demonstrated that the C-terminal region of TLQP-21 is the sequence retaining the full biological activity (Cero et al., 2014) and likely also the region primarily involved in the binding and activation of its receptor/s, we decided to use 10 μM JMV5656 for the following patch-clamp experiments.

### JMV5656 Effects on N9 Cells Transmembrane Currents

**FIGURE 2:**
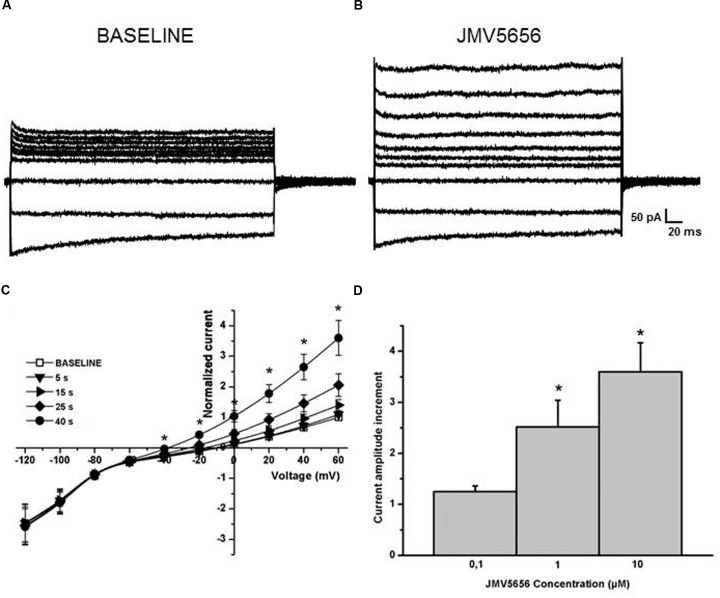
**Effects of JMV5656 superfusion on N9 cells.** **(A,B)** Representative families of currents traces acquired from N9 cells through a step protocol from –120 to +60 mV applied form a holding potential of -80 mV in control conditions **(A)**, and every five seconds from the opening of the perfusion with JMV5656 up to 40 s **(B)**. **(C)** Current/voltage relationship in the presence of JMV5656. In the plot, the currents amplitude is presented as normalized relative to the baseline current for each cell. Normalized currents measured were 0.92 ± 0.09 and 3.6 ± 0.5 after 40 s perfusion with extracellular solution and 10 μM JMV5656 (*n* = 25), respectively. Data are presented as mean ± SE. **(D)** Bars graph showing the dose-response effect of the perfusion of JMV5656 measured at +60 mV (*n* = 8, 8 and 25 for 0.1 μM, 1 μM, and 10 μM, respectively. ^∗^*p* < 0.05 vs. 0.1 μM JMV5656).

The perfusion of 10 μM JMV5656 induced an increase of about threefold in the total outward currents in 79% of cells tested (41 cells out of 52). The total outward currents increased to 33.3 ± 7.8 pA/pF, from a value of 10.9 ± 2.7 pA/pF measured at baseline condition at 60 mV (**Figures 2A–C**). Moreover, the value of the reversal potential hyperpolarized from -19.6 ± 3.3 mV to –35.7 ± 8.3 mV (*p* < 0.05) after 40 s of the peptide perfusion (**Figure 2C**), time in which the increase of the outward currents reached a plateau, before returning to baseline (**Supplementary Figure S3**). This effect was concentration-dependent (**Figure 2D**).

### JMV5656 Activated Potassium Currents in N9 Cells

**FIGURE 3:**
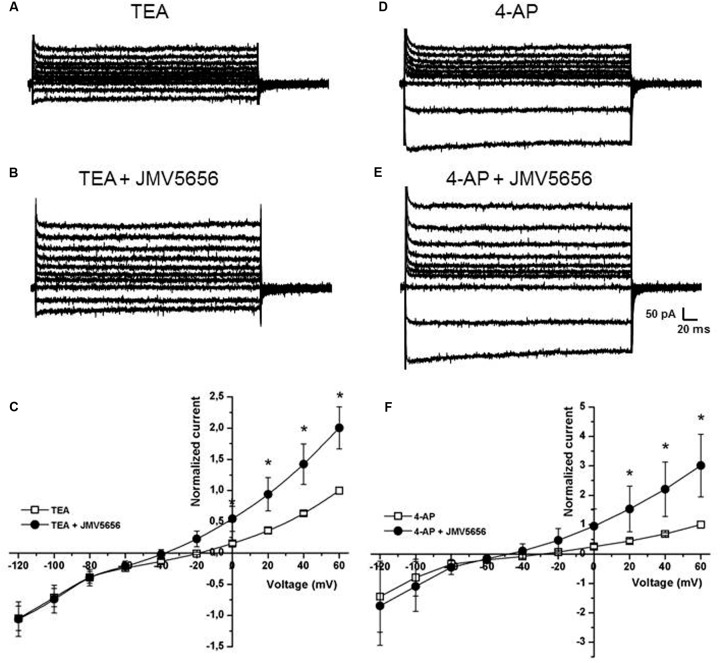
**Effects of TEA and 4-AP on the superfusion of JMV5656 in N9 cells.** **(A,B)** Representative families of current traces recorded in N9 cells perfused with extracellular solution containing TEA **(A)** and after 40 s of perfusion with TEA plus JMV5656 peptide **(B)**. **(C)** Current/voltage relationship at different time points from the opening of the perfusion of extracellular solution containing TEA plus JMV5656. Currents are normalized to the value of the baseline current recorded at +60 mV. Empty symbols indicates the presence of TEA, filled symbols the presence of TEA and JMV5656. **(D,E)** Representative families of current traces recorded in N9 cells perfused with extracellular solution containing 4-AP **(D)** and after 40 s of perfusion with 4-AP plus JMV5656 peptide **(E)**. **(F)** Current/voltage relationship at different time points from the opening of the perfusion of extracellular solution containing 4-AP plus JMV5656. Currents are normalized to the value of the baseline current recorded at +60 mV. Empty symbols indicate the presence of 4-AP, filled symbols the presence of 4-AP and JMV5656. Data are expressed as mean ± SE. ^∗^*p* < 0.05 vs respective baseline values (*n* = 5 in all conditions).

The results obtained, in particular the hyperpolarization of the reversal potential, suggested that the peptide could activate a potassium current. Consequently, TEA, a non-selective blocker of voltage-dependent and Ca^2+^-activated potassium (K^+^) channels, was added to the extracellular solution at 10 mM in order to determine whether JMV5656 could influence K^+^ currents. First, we recorded current traces in N9 cells perfused with the extracellular solution containing 10 mM TEA alone (**Figure 3A**). When TEA was subsequently administered in combination with 10 μM JMV5656, the outer membrane currents increased by 2.00 ± 0.3-fold (from 14.3 + 6.4 pA/pF in TEA alone to 26.5 ± 8.8 pA/pF in TEA+JMV5656, values measured at 60 mV; *p* < 0.05) (**Figures 3B,C**). The ability of TEA to attenuate JMV5656-stimulated outward currents so significantly suggested an involvement of K^+^ channels activation in the N9 cells response to JMV5656 stimulation. In order to discriminate whether voltage-gated K^+^ channels were involved in the response, we perfused the cells with 5 mM 4-AP, a broad-spectrum blocker of voltage-gated K^+^ channel (**Figure 3D**). Interestingly, 4-AP did not inhibit the effect of JMV5656, which was still capable to induce a 3.0 ± 1.1-fold increase of outward currents (**Figures 3E,F**).

### JMV5656 Activates Calcium-Dependent Potassium Channels

**FIGURE 4:**
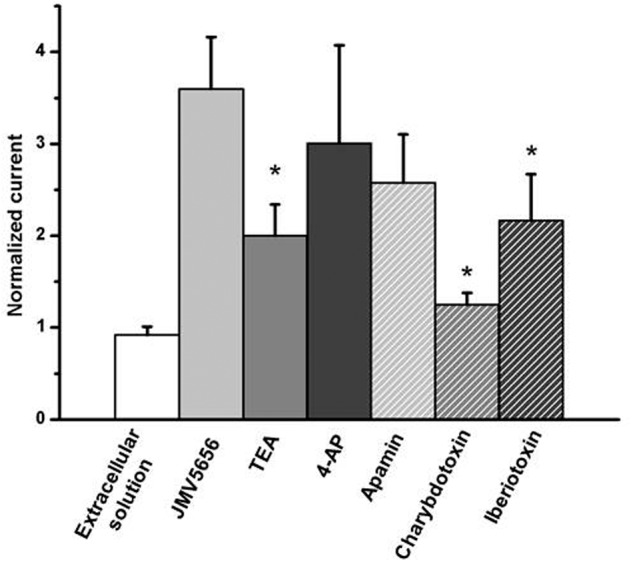
**Effect of the perfusion of extracellular solutions containing JMV5656 plus potassium channels blockers.** Data are expressed as current amplitude recorded at +60 mV after 40 s of perfusion and normalized to the value of the baseline current recorded at the same voltage membrane. Data are expressed as mean ± SE (*n* = at least eight cells for each condition) ^∗^*p* < 0.05 vs. JMV5656

Since JMV5656 dose-dependently boosted a significant increase of intracellular calcium levels in N9 cells (**Figure 1**), we focused our attention on the possible involvement of calcium-activated potassium channels in its mechanism of action. To ascertain whether K_Ca_2 channels were engaged in this JMV5656 activity, N9 cells were perfused with apamin, a drug that blocks K_Ca_2.2 at 200 pM and K_Ca_2.1 and K_Ca_2.3 at nM concentrations (Wulff et al., 2007). Interestingly, 100 nM apamin could not prevent the rise of total outward currents stimulated by JMV5656 perfusion: when measured at +60 mV, the current amplitude increased about 2.5 ± 0.5-fold (*p* < 0.05) from 18.9 ± 6.7 pA/pF in the apamin group to 45.8 ± 10 pA/pF in the apamin + JMV5656 (**Figure 4**). These data suggested that K_Ca_2 channels were not primarily involved in JMV5656 mechanism of action. Application of charybdotoxin (CTX, 100 nM), a K_Ca_1.1, K_Ca_3.1, and voltage-gated Kv1.3 channel’s blocker (Gao and Garcia, 2003), instead, yielded a 1.25 ± 0.13-fold higher variation in the outer currents compared to controls (**Figure 4**), suggesting that K_Ca_1.1 or K_Ca_3.1 channels could be associated to the JMV5656 mechanism of action. We ruled out the involvement of Kv1.3 channels since they are also inhibited by TEA and 4-AP (Comes et al., 2013). It is worthy to remember that K_Ca_3.1 channels are insensitive to TEA, which instead, blocks K_Ca_1.1 (Tricarico et al., 2013). When N9 cells were perfused with an extracellular solution containing 100 nM iberiotoxin (IbTX) that selectively inhibits K_Ca_1.1 channels (Gao and Garcia, 2003), JMV5656 was still capable of stimulating a partial increase of the total outward currents (2.2 ± 0.5-fold), suggesting that K_Ca_1.1 channels could be involved in the JMV5656 activated pathway (**Figure 4**).

Thus, these results suggested that (i) JMV5656-stimulated potassium outward current is not driven by a single type of calcium activated K^+^ channel, and (ii) that both K_Ca_1.1 and K_Ca_3.1 might be recruited.

### JMV5656 Could Be Responsible for K_Ca_3.1 Channels Activation

NS6180, which has been recently identified as a novel K_Ca_3.1 channel’s inhibitor (Strøbæk et al., 2013), was used to investigate the potential involvement of K_Ca_3.1 channels in the effects of JMV5656 perfusion. When NS6180 at a concentration of 250 nM was added to the superfusion solution containing the peptide, the outward current declined of the 94,6 ± 4.6 % (*n* = 6, data not shown), while no effect of the drug was visible on the baseline currents (**Supplementary Figure S4**).

**FIGURE 5:**
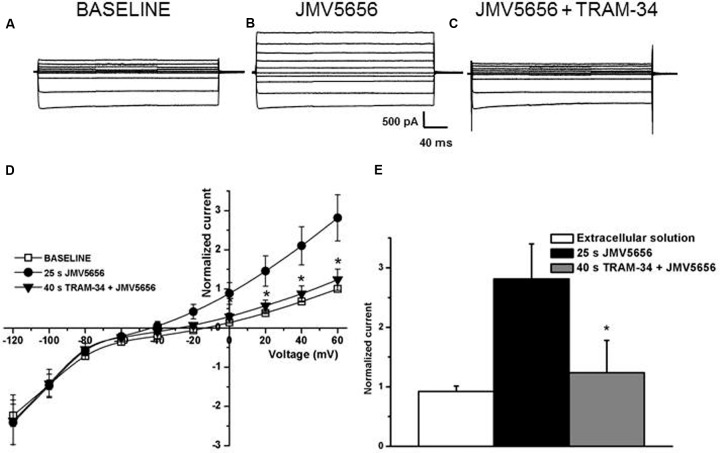
**Effects of TRAM-34 on N9 cells superfused with JMV5656.** **(A–C)** Representative families of current traces recorded in N9 cells before **(A)** and after 25 s **(B)** of perfusion with JMV5656 peptide and after 40 s of perfusion with JMV5656 peptide plus TRAM-34 **(C)**. **(D)** Current/voltage relationship of baseline (empty symbols), 25 s of JMV5656 perfusion (filled circles) and 40 s of TRAM-34 plus JMV5656 perfusion (filled triangles). Currents are normalized on the value of baseline current recorded at +60 mV (*n* = 19). **(E)** Bar graph representing normalized current after 40 s of perfusion. Data have been obtained in three independent experiments. Data are expressed as mean ± SE.

To further characterize the involvement of K_Ca_3.1 channels in the JMV5656 action, we measured the effects of TRAM-34, one of the most recognized selective blocker of K_Ca_3.1 channels (Wulff et al., 2000). In this instance, N9 cells were (i) first superfused either with the extracellular solution alone (**Figure 5A**) or with the extracellular solution containing 10 μM JMV5656 for 25 s (**Figure 5B**), an interval of time sufficient for JMV5656 effects to become significant (the outer current increased from 15.0 ± 4.2 pA/pF to 37.5 ± 10.1 pA/pF; *p* < 0.05), and (ii) thereafter switched to a solution containing JMV5656 and 2 μM TRAM-34 (**Figure 5C**). The current amplitude decreased significantly (19.0 ± 8.6 pA/pF) (**Figures 5D,E**).

**FIGURE 6:**
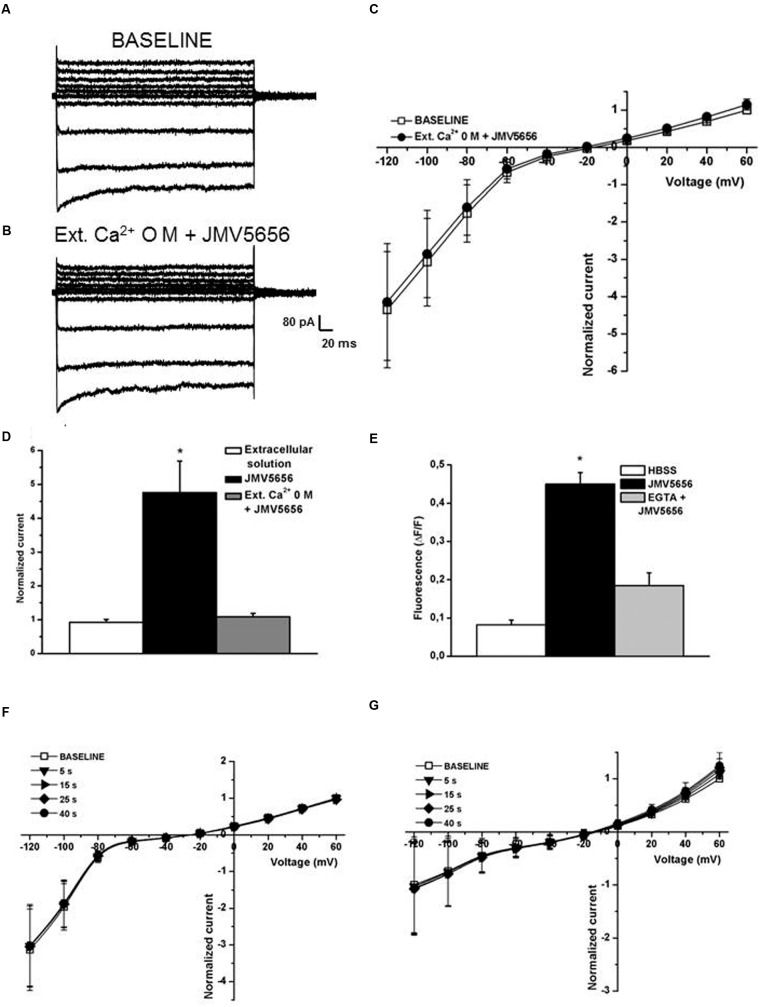
**Extracellular and intracellular Ca^2+^ dependence of JMV5656 effect on K_Ca_3.1 channels.** **(A,B)** Representative families of currents traces recorded in N9 cells perfused with the regular extracellular solution **(A)** and after 40 s of extracellular solution containing 0 mM Ca^2+^ and JMV5656 **(B)** in a condition of intracellular free calcium concentration of 100 nM. **(C)** Current/voltage relationship during perfusion with the extracellular solution (baseline, empty symbols) and perfusion containing JMV5656 (filled symbols) but lacking of Ca^2+^. **(D)** Normalized current amplitude recorded at +60 mV after 40 s of perfusion with extracellular solution (*n* = 11), JMV5656 (*n* = 9), JMV5656 without extracellular Ca^2+^ (*n* = 15). **(E)** Cytosolic calcium mobilization, expressed as variation in fluorescence intensity, obtained in N9 cells stimulated with the vehicle only (HBSS), with JMV5656 and with JMV5656 in presence of 1 mM EGTA to chelate the extracellular calcium. **(F)** Current/voltage relationship obtained in presence of JMV5656 in the extracellular solution and BAPTA 5 mM in the intracellular one (*n* = 5). **(G)** Current/voltage relationship obtained in presence of JMV5656 in the extracellular solution and 3 μM free intracellular calcium (*n* = 16); recording were made at selected time points (5, 15, 25, and 40 s). ^∗^*p* < 0.05 vs control group. All data have been obtained in three independent experiments.

Extracellular calcium is crucial for the activation of K_Ca_3.1 channels (Ferreira and Schlichter, 2013) and indeed our data indicate that JMV5656 failed to activate the outward currents when the extracellular environment was devoid of Ca^2+^ (**Figures 6A–D**). In fact, the outward currents density measured at +60 mV was similar before (11.2 ± 4.5 pA/pF) and after JMV5656 superfusion (14.4 ± 7.1 pA/pF). Accordingly, when extracellular calcium is chelated by the presence of EGTA 1 mM, also the mobilization of the intracellular calcium is very low and did not significantly differed from the control condition, blunting the effect of JMV5656 (**Figure 6E**).

To further confirm a substantial involvement of K_Ca_3.1 beside K_Ca_1.1 in the peptide response, we reasoned on the potassium channel calcium sensitivity. Outward currents were recorded in presence of 3 μM free intracellular calcium concentration, a condition at which K_Ca_3.1 is fully activated, while K_Ca_1.1 is not. In this scenario, we observed that the effect of JMV5656 was gone, despite the reversal potential of the total transmembrane currents was shifted in the hyperpolarized direction as it appeared after the activation of the outward current by the peptide (**Figure 6F**). Finally, in presence of BAPTA as intracellular calcium chelator, JMV5656 failed to activate K_Ca_ currents (**Figure 6G**).

### Inhibition of K_Ca_3.1 Channels mRNA Levels in N9 Cells

**FIGURE 7:**
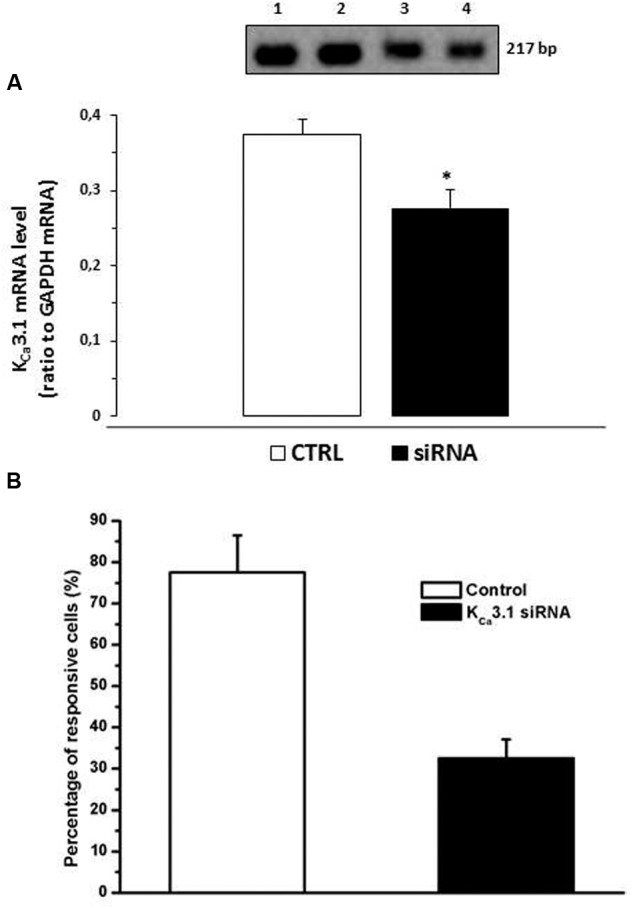
**Effect of K_Ca_3.1 siRNA on N9 cells superfused with JMV5656.** **(A)** Bars graph showing that K_Ca_3.1 mRNA levels were significantly reduced after transfection with 50 nM siRNA for 24 h. The inset shows representative PCR: lane 1 N9; lane 2 N9 transfected with siRNA negative control; lane 3–4 N9 transfected with K_Ca_3.1 siRNA. **(B)** Bar graph showing that in presence K_Ca_3.1 siRNA the percentage of N9 cells responsive to JMV5656 was reduced from 79% (control cells) to 33% (siRNA transfected cells) (*n* = 9). Data are the mean ± SE of results obtained in three independent experiments. ^∗^*p* < 0.05 vs control group.

To confirm the role of K_Ca_3.1 channels in JMV5656 effects, we used specific siRNA to reduce the mRNA levels of this channel in N9 cells. Transfection of K_Ca_3.1 siRNA duplex significantly reduced (*p* < 0.05) K_Ca_3.1 mRNA levels (**Figure 7A**). Moreover, after silencing K_Ca_3.1 channels, there was a significant decrease in the number of cells responsive to JMV5656 stimulation in terms of activation of outward currents (**Figure 7B**). Cells responding to JMV5656 decreased from 79% in control group to 33% in the siRNA group. Among the responsive cells, the outward current increase was confirmed to be of about 3.2 ± 0.7-fold. These results support the hypothesis of a K_Ca_3.1 involvement in the JMV5656-induced effect.

## Discussion

The diversity of known biological- and medically relevant activities for TLQP-21(VGF^556-576^) is expanding rapidly. Accordingly, this study investigates modulation of selected transmembranal ionic currents as an element of TLQP-21 intracellular signaling in the microglial cell model. TLQP-21 was initially immunopurified from rat brain and further immunolocalized in gastric tissue as well as in adrenergic neurons (Hahm et al., 1999; Bartolomucci et al., 2006; Brancia et al., 2010). The known biological effects of TLQP-21 are diverse. Although VGF knockout mice are smaller and thinner compared to their wild type littermates (Salton et al., 2000), TLPQ-21 was shown to induce anorexic effects, to activate lipolysis and modulate inflammatory pain, and to blunt obesity induced by diet (Bartolomucci et al., 2006, 2009; Rizzi et al., 2008). The biological effects of TLQP-21 are receiving increasing attention, but the specific receptor(s), which mediate its effects are still a matter of considerable debate (Chen et al., 2013; Hannedouche et al., 2013); moreover, there is little information about the TLQP-21 mechanism of action. Our results demonstrate that TLQP-21 like JMV5656 (i.e., TLQP-21^9-21^) increased intracellular calcium levels in N9 microglia cells. We have decided to perform our experiments using the N9 immortalized mouse microglia cells, which were previously demonstrated to be a suitable model for studies on microglia (Bureau et al., 2008; Wang et al., 2008) and might be a valuable alternative to primary mouse microglia culture for use in pharmacological and toxicological investigations. Microglial cells are known to be the immune effector cells in the brain (Streit, 2004) and the first line of defense against pathogens and acute or chronic brain injuries. Reportedly, neurodegenerative diseases, stroke and tumor invasion, induce an activation of microglia cells that enclose damaged and dead cells and remove cellular debris from the area, functioning as phagocytic macrophages. Microglial cells are responsible for the release of a variety of pro-inflammatory mediators such as cytokines, reactive oxygen species, complement factors, neurotoxic secretory products, free radical species, and nitric oxide (NO) which could contribute to both neuronal dysfunction and cell death (Griffin et al., 1998).

In N9 microglia cells, we have found that JMV5656 possesses the same biological activity with slightly higher potency than TLQP-21 in its ability to enhance [Ca^2+^]_i_. These results are in agreement with the study of Cero et al. (Cero et al., 2014) who demonstrated that the hot spots for the biological activity of the TLQP-21 are in its C-terminus and that the retention of its last thirteen amino acids is sufficient to have a peptide with a comparable biological activity. In terms of quantification and how high JMV5656 raised intracellular Ca^2+^, we could speculate from what is known from the literature. In N13, a cell line immortalized from primary mice microglial cell and very similar to N9, the basal intracellular calcium concentration was found to be about 150–200 nM (Ferrari et al., 1996). Since when we stimulated with 10 μM JMV5656 the increase in calcium level was of about 40%, we expect that the free calcium concentration in this condition might be about 210–280 nM.

In microglia, the elevation of cytosolic calcium levels is necessary for cytokine induction (Hoffmann et al., 2003) and cellular activation. Moreover, previous studies reported a tight association between intracellular calcium-dependent signaling and Ca^2+^-dependent potassium channels activity (Stocker, 2004). For example, Ca^2+^-dependent potassium channels, such as K_Ca_1.1, K_Ca_3.1, and K_Ca_2.3 seemed to be linked in particular to microglial activation processes (Bordey and Spencer, 2003; Schlichter et al., 2010).

Indeed, our data show that JMV5656 was responsible for the activation of an outward K^+^ current which is calcium dependent as revealed by charybdotoxin and iberiotoxin sensitivity. We speculate that the general role of these potassium channels is to provide a membrane polarizing influence (viz. potassium efflux) which offsets the depolarizing action of calcium elevation, and indirectly to maintain the driving force for optimal calcium increase in the cytosol. Moreover, the calcium-activated potassium channels help to regulate the volume of microglial cells during migration. The changes of shape and volume seem to be a prerequisite for cell migration. It is possible that in microglia, calcium-activated potassium currents cause wrinkling of the cell body, as proposed by Schwab (2001). This change of shape can promote the migration, facilitating the retraction of the rear part of the cell.

Interestingly, our results suggest that JMV5656 mainly, even not specifically, activates K_Ca_3.1 currents. In fact, while apamin, a blocker of K_Ca_2 when used in the nanomolar range, was not able to prevent the increase in outward K^+^ currents, charybdotoxin that inhibits K_Ca_1.1, K_Ca_3.1 channels (Gao and Garcia, 2003) and iberiotoxin, that inhibits K_Ca_1.1 (Salton et al., 2000), were capable of blunting (the former in a complete way, the second partially) the ability of JMV5656 to activate outward K^+^ currents. Further details were gained by the use of NS6180 and TRAM-34, which allowed proving that mostly the intermediate-conductance Ca^2+^-activated K^+^ channels K_Ca_3.1 were involved in the effects of JMV5656 perfusion. Moreover, K_Ca_3.1 activation depends on the presence of extracellular calcium and this channel activity is not induced by several stimuli that release Ca^2+^ from intracellular stores but which do not stimulate Ca^2+^ influx (Cruse et al., 2006), and our results are in line with this knowledge. To strengthen the hypothesis of a prevalent contribution of K_Ca_3.1 channels than K_Ca_1.1 in the peptide response, we reasoned on their respective calcium sensitivity. The typical intracellular solution for whole cell measurements allowed us to have an free calcium concentration of 100 nM, that is below the threshold of the calcium-dependent activation of both K_Ca_3.1 and K_Ca_1.1. The former channel relies on the use of calmodulin as Ca^2+^ sensor, thus shows an intrinsically high affinity for calcium, while for the second, the calcium bowl requires higher calcium concentration to activate the protein. Considering that JMV5656 induced a 40% increase of free [Ca^2+^]_i_ (**Figure 1B**), the intracellular calcium level reached after the peptide perfusion was compatible with the activation of K_Ca_3.1 (Joiner et al., 1997; Ferreira and Schlichter, 2013) but not of the K_Ca_1.1 (Rothberga and Magleby, 2000). Furthermore, a [Ca^2+^]_i_ of 3 μM, fully activates K_Ca_3.1, while the full activation of K_Ca_1.1 required 10 μM [Ca^2+^]_i_. Indeed, in 3 μM free calcium, JMV5656 failed to activated a large outward current, consistent with the prevalence of K_Ca_3.1 already activated (hypothesis confirmed also by the hyperpolarization of the V_rev_ already significant before the peptide perfusion). Finally, the partial silencing of the mRNA for K_Ca_3.1 reduced the number of cells responsive to JMV5656.

It is interesting at this point at least TO speculate what the sources for the JMV565 triggered Ca^2+^ influx could be. The data obtained in presence of BAPTA in the intracellular solution suggested a coupling between the calcium source and the calcium sensor (K_Ca_) in the nanometers range (Fakler and Adelman, 2008). In fact, in accordance with this interpretation, BAPTA, with its 150 times faster calcium binding rate compared to EGTA, thus more effective in preventing calcium diffusion, interfered with the outcome of JMV5656. Extracellular calcium was necessary to activate JMV5656 response, thus one may think that calcium channels on the plasma membrane may be the primum movens required to trigger the K_Ca_ activation which may be found in their very close proximity. In microglial cells, L-type calcium channels are indeed present and preliminary data (not shown) from our lab indicated that nifedipine (10 μM) blunted the increase of the outward current JMV5656-mediated (only 40% respect to the baseline), suggesting a potential involvement of L-type calcium channel in the activation of the K_Ca_. But, since the outward current required at least 25 s of peptide perfusion to manifest, it would be hard to think at a direct effect of the peptide on the L-type calcium channel opening, but quite reasonably this delay is more consistent with the generation of second messengers that may affect the Ca^2+^ channel activity.

Thus, we suggest that JMV5656 acting on its receptor(s) generates an increase in cytosolic calcium that, together with the depolarization, may open calcium channels (L-type?) found on the plasma membrane of the N9 cells. The calcium ions entered through this route might favor the activation of an outward potassium current mediated mainly by K_Ca_3.1 found in their nanometers range proximity.

The ability of JMV5656 (and TLQP-21) to stimulate microglia cells is an interesting finding that might help for the development of antagonist as new therapeutic tools for the care and the treatment of neuropathic pain. In fact, it has been reported that injection of TLQP-21 into the hind paw of mice resulted in hypersensitivity in both control conditions and in a model of inflammatory pain (Rizzi et al., 2008). In the dorsal horn, stimulation of microglia by TLQP-21 could lead to production and secretion of cytokines responsible for the activation of sensory neurons. Recently the gC1qR and C3AR1 complement protein receptors, have been proposed to be involved in the TLQP-21 mechanism of action (Chen et al., 2013; Hannedouche et al., 2013).

## Conclusion

By measuring the outward K^+^ currents, we were able to demonstrate that by interacting with its cellular receptors, JMV5656, a derivative of TLQP-21, was capable to stimulate increase in intracellular calcium, which activated, even not in exclusively manner, K_Ca_3.1 channels in N9 microglia cells. The opening of K_Ca_3.1 channels is important for the hyperpolarization of the plasma membrane which allows maintaining the driving force for Ca^2+^ to entry from the extracellular environment and an optimal replenishment of intracellular Ca^2+^ stores.

## Author Contributions

IR, AT: Substantial contribution to the design of the work, interpretation of the data, drafting the work, final approval of the version to be published, agreement to be accountable for all the aspects of the work. AB, LM, LR, EB: Substantial contribution to the acquisition, analysis of the work, final approval of the version to be published, agreement to be accountable for all the aspects of the work. RP, RO, J-AF, PV, JM, VL: Substantial contribution to the interpretation of the data, final approval of the version to be published, agreement to be accountable for all the aspects of the work.

## Conflict of Interest Statement

The authors declare that the research was conducted in the absence of any commercial or financial relationships that could be construed as a potential conflict of interest.

## Footnotes

1 http://maxchelator.stanford.edu/CaEGTA-TS.htm
