# Supplementary figures and images for: JMV5656, A Novel Derivative of TLQP-21, Triggers the Activation of a Calcium-Dependent Potassium Outward Current in Microglial Cells
Source: Front Cell Neurosci. 2017 Feb 23;11:41. doi: 10.3389/fncel.2017.00041 (PMC5322282; doi:10.3389/fncel.2017.00041)

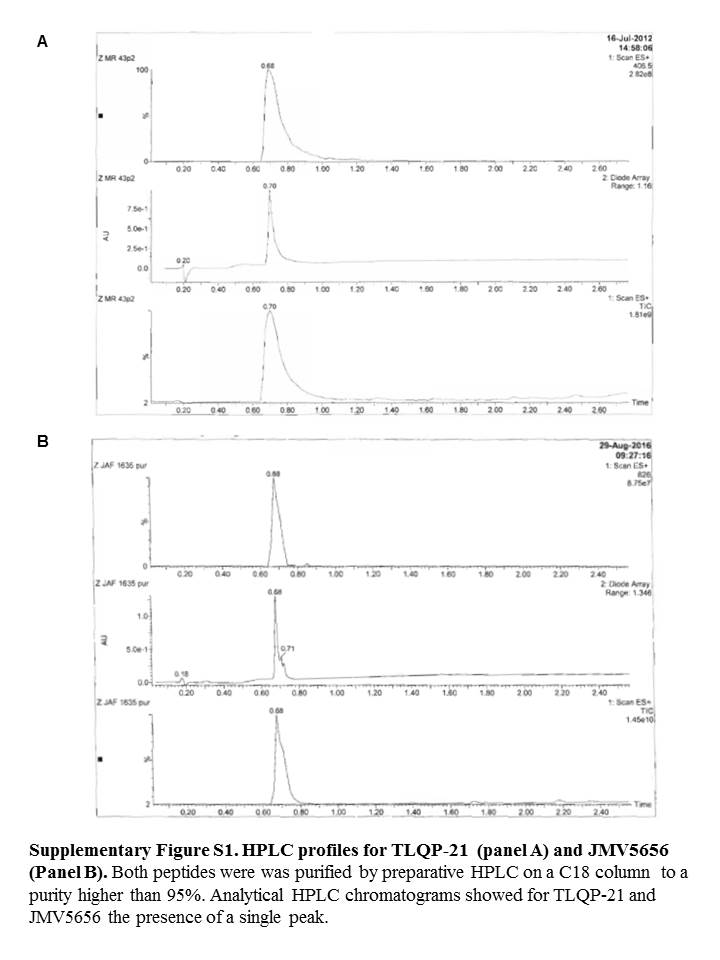

Supplement: Supplementary file 1 [file Image_1.jpg]

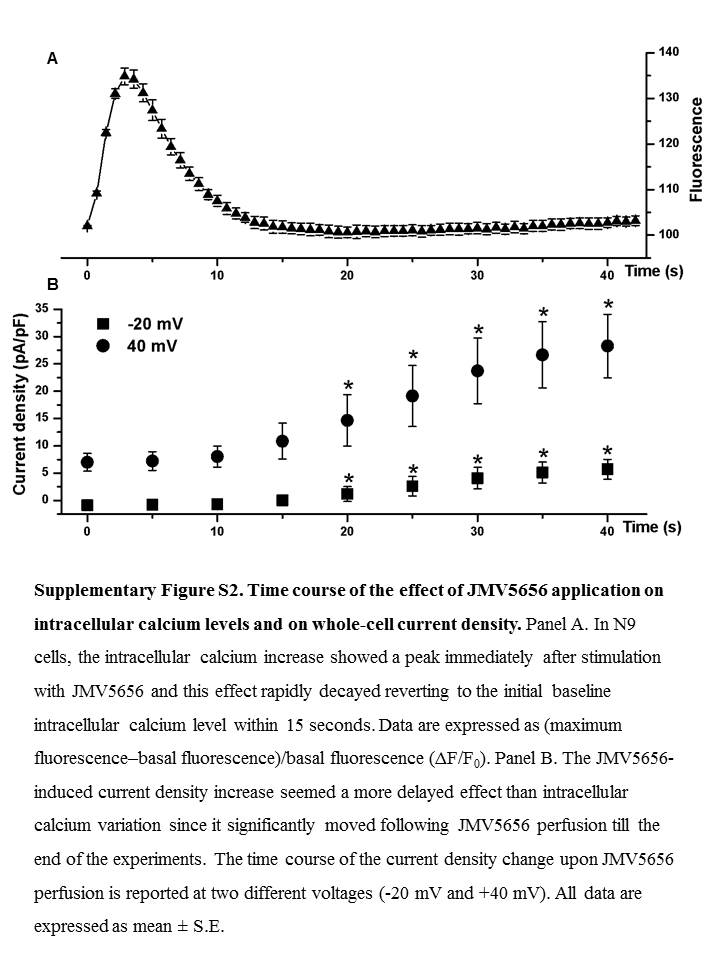

Supplement: Supplementary file 2 [file Image_2.jpg]

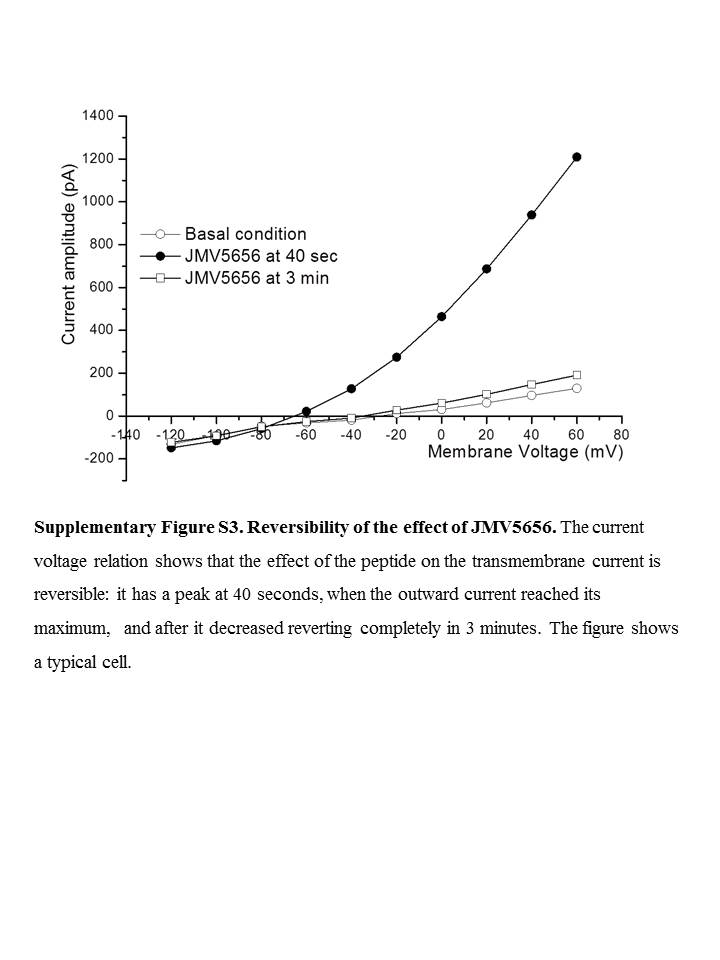

Supplement: Supplementary file 3 [file Image_3.jpg]

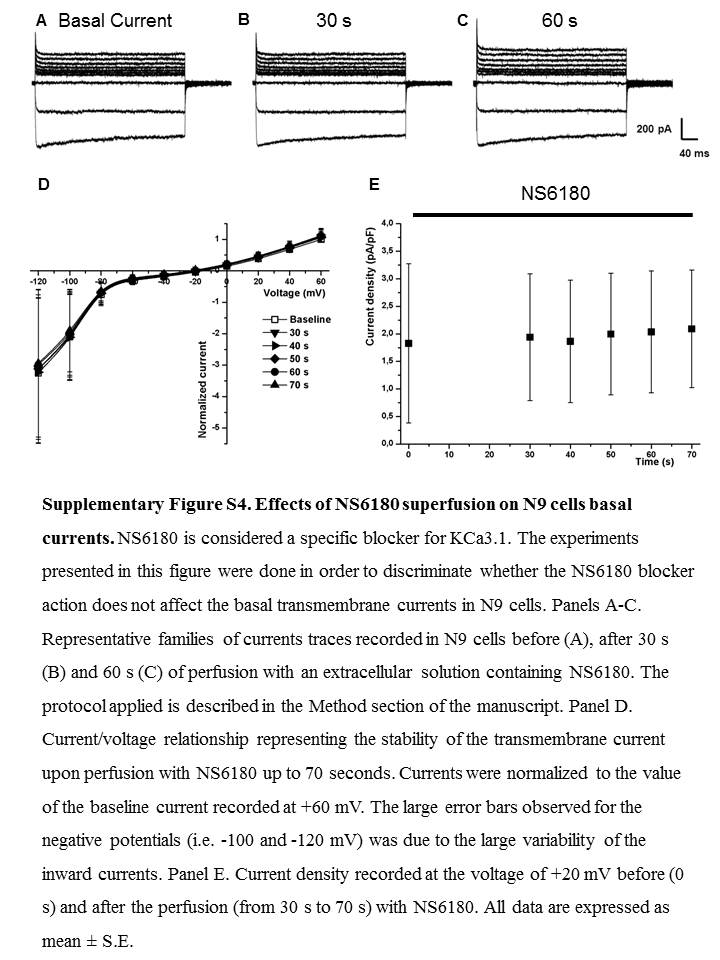

Supplement: Supplementary file 4 [file Image_4.jpg]
